# Exploring the mediating role of meaning in life and psychological resilience in the relationship between physical exercise and sleep quality among Chinese secondary school students

**DOI:** 10.3389/fpsyg.2025.1714320

**Published:** 2025-12-15

**Authors:** Lihua Yao, Yang Wu, Kelei Guo, Jun Xiang

**Affiliations:** 1 School of Physical Education, Shangqiu Normal University, Shangqiu, China; 2 School of Recreational Sports and Tourism, Beijing Sport University, Beijing, China; 3 School of Physical Education and Health, Zhaoqing University, Zhaoqing, China

**Keywords:** physical exercise, sleep quality, sense of meaning in life, psychological resilience, junior high school students

## Abstract

**Introduction:**

The persistent decline in adolescents’ sleep quality has become a significant public health issue affecting their physical and mental well-being as well as academic development, necessitating the exploration of effective intervention strategies.

**Objective:**

To investigate the relationship between physical exercise and sleep quality among middle school students, and to examine the chain-mediated role of life meaning and psychological resilience in this relationship.

**Methods:**

A stratified cluster sampling method was employed to conduct a questionnaire survey among 1,579 junior high school students (833 males, 746 females). Measurement tools included the Revised Physical Exercise Scale, the Pittsburgh Sleep Quality Index, the Sense of Meaning in Life Scale, and the psychological resilience subscale from the Positive Adolescent Development Scale. Data analysis employed Pearson correlation analysis supplemented by bias-corrected percentile bootstrap methods.

**Results:**

(1) Physical exercise showed a significant positive correlation with sleep quality (*R* = 0.268, *p* < 0.001), with a significant direct effect (*β* = 0.028, *p* < 0.001); (2) Physical exercise showed significant positive correlations with both life meaning (*β* = 0.517, *p* < 0.001) and psychological resilience (*β* = 0.132, *p* < 0.001). Life meaning was significantly positively correlated with psychological resilience (*β* = 0.120, *p* < 0.001) and sleep quality (*β* = 0.007, *p* < 0.001). Psychological resilience was significantly positively correlated with sleep quality (*β* = 0.079, *p* < 0.001); (3) Chain mediation analysis revealed that both life meaning and psychological resilience significantly mediated the relationship between physical exercise and sleep quality, involving three pathways: physical exercise → life meaning → sleep quality (mediation effect accounts for 10.35%), physical exercise → psychological resilience → sleep quality (mediation effect accounts for 34.48%), physical exercise → sense of meaning in life → psychological resilience → sleep quality (mediation effect accounts for 17.24%).

**Conclusion:**

Physical exercise not only directly improves sleep quality among secondary school students but also exerts indirect positive effects by enhancing sense of meaning in life and psychological resilience. This study provides empirical support for understanding the mechanism linking physical exercise, psychological resources, and sleep quality. Practically, schools and educational authorities can embed regular, diverse physical exercise programs into curricula and integrate activities fostering life meaning and psychological resilience to form a synergistic intervention mechanism, thereby promoting adolescent mental health and high-quality sleep. Policy makers can accordingly incorporate psychological adaptability and life meaning education into campus health promotion and youth sports development plans, providing comprehensive strategies to enhance adolescent sleep quality.

## 1 Introduction

Adolescence is a critical stage of rapid physical and mental development for young people. Adequate and high-quality sleep is essential for supporting normal physiological functions, mental health, and cognitive development. Chronic sleep deprivation not only impairs learning and memory abilities but is also closely associated with various health issues, including emotional disorders, obesity, increased risk of cardiovascular and cerebrovascular diseases, and reduced adaptability (Kansagra, 2020). However, in recent years, influenced by factors such as accelerated social rhythms and increased academic burdens, sleep deprivation among Chinese middle school students has become widespread and is intensifying. Surveys indicate that the prevalence rate of sleep disorders among Chinese middle school students reaches 24.0%, significantly higher than in some other countries. The China National Mental Health Report (2019–2020) points out that 90.8% of junior high school students do not get sufficient sleep (Xu et al., 2021). Although government and education authorities have implemented policies mandating adequate daily sleep for students at all educational levels (Cheng et al., 2022), real-world enforcement remains inadequate, necessitating the exploration of effective improvement strategies.

Extensive empirical research demonstrates a significant positive correlation between physical exercise and sleep quality, with moderate physical activity improving adolescents’ sleep outcomes (Kansagra, 2020). However, the psychological mechanisms through which physical exercise influences sleep quality remain poorly understood. A notable theoretical gap exists in the literature regarding the mediating roles of life meaning and psychological resilience. Life meaning, by providing clear purpose and value to life, can reduce psychological stress and enhance sleep. Psychological resilience, meanwhile, strengthens individuals’ ability to cope with adversity, thereby mitigating the disruptive effects of negative emotions on sleep. These two factors may form a sequential chain of effects, transmitting the positive impact of physical exercise to improved sleep quality. The theoretical value of this chain-mediated model lies in its integration of a positive psychology perspective while elucidating the underlying psychological pathways through which physical exercise influences sleep, offering a novel explanatory framework for adolescent sleep health research. Based on this, the present study systematically analyzes the relationship between physical exercise and sleep quality among Chinese junior high school students, focusing on examining the sequential mediating role of life meaning and psychological resilience in this relationship. The study aims to: (1) validate the positive effects of physical exercise on sleep quality; (2) reveal the underlying transmission mechanisms of positive psychological traits; (3) provide empirical support for developing more theoretically grounded and practically guided adolescent sleep intervention strategies.

## 2 Literature review and research hypotheses

### 2.1 Physical exercise and sleep quality

Physical exercise refers to bodily activities that utilize physical training and exercise loads as means, encompassing fitness training, recreational leisure, health maintenance and rehabilitation, as well as mental and cognitive development. These activities aim to enhance physical fitness, promote mental and physical well-being, and improve and sustain bodily capabilities (Herbert, 2022). Sleep constitutes a fundamental physiological process essential for sustaining human life and health. Research indicates a significant association between physical exercise and sleep quality (Min et al., 2021). First, scholars note that exercise elevates body temperature, which is regulated by the hypothalamus to facilitate efficient heat dissipation. This process helps prolong participants’ slow-wave sleep and stabilize deep sleep states. Concurrently, exercise stimulates melatonin secretion, thereby improving sleep quality (Korkutata et al., 2025). Second, studies indicate that regular physical activity helps individuals maintain positive emotions and sound mental states. Moderate-intensity aerobic exercise, in particular, is believed to effectively unblock meridians and optimize organ function. This enables practitioners to achieve a physiological state of mental calmness, physical relaxation, and natural breathing, prompting the pineal gland to release more pineal hormone (melatonin), ultimately improving sleep quality (Skillett, 2023). Finally, studies reveal that regular exercisers exhibit optimal sleep quality, while non-exercisers experience the poorest. Both general and regular exercise demonstrate particularly significant improvements in sleep. Research concludes that extracurricular physical exercise is a key factor influencing students’ sleep quality, with physical activity widely regarded as an important positive factor promoting student sleep quality (Li et al., 2022; Lin et al., 2022). In summary, it can be inferred that physical exercise is closely related to sleep quality among secondary school students. Based on this, Hypothesis 1 is proposed: Physical exercise is positively correlated with sleep quality.

### 2.2 Mediating role of sense of meaning in life

One of the mediating mechanisms in this study is the mediating role of sense of meaning in life. Sense of meaning in life plays a crucial mediating role between physical exercise and sleep quality. Meaning in life reflects an individual’s understanding and comprehension of life’s significance, encompassing awareness of life’s purpose, mission, and primary goals (Bayram and Artan, 2024). First, research indicates a significant positive association between physical exercise and meaning in life among college students, with exercise positively predicting their levels of meaning in life (Zhao and Yin, 2024). Furthermore, the flow experiences generated during physical exercise and the secretion of endorphins in the brain can also enhance an individual’s sense of meaning in life to a certain extent (Cullen, 2023; Martín-Rodríguez et al., 2024). Existing research also indicates that increasing investment in physical exercise contributes to enhancing one’s sense of meaning in life. Physical exercise promotes the development of personality traits, which in turn influences an individual’s sense of meaning in life (Guo et al., 2024). Furthermore, exercise psychology research suggests that intensifying physical activity is an effective pathway to elevate life meaning levels. Physical exercise helps shape more positive attribution styles, explanatory styles, and humor styles, thereby enhancing optimistic personality traits and ultimately promoting life meaning (Khan and Subhan, 2023). Moreover, as a protective resource for mental health, individual characteristics (such as life meaning) can moderate the relationship between environmental factors and psychological outcomes (like sleep quality). Life meaning significantly negatively predicts subjective sleep quality and academic stress—that is, stronger life meaning correlates with better sleep quality and lower academic stress (Ge, 2025). Scholars’ research also emphasizes that a sense of life meaning helps individuals more keenly perceive and utilize various favorable factors, including environmental ones, thereby promoting better sleep conditions (Prakash et al., 2020; Hooker et al., 2018). In summary, it can be inferred that high levels of life meaning are typically associated with a lower risk of sleep problems. Physical exercise not only directly improves sleep quality but also exerts an indirect positive influence through the mediating pathway of enhancing life meaning. Based on this, Hypothesis 2 is proposed: Life meaning mediates the relationship between physical exercise and sleep quality.

### 2.3 Mediating role of psychological resilience

One of the mediating mechanisms in this study is the mediating role of psychological resilience. Psychological resilience plays a crucial mediating role between physical exercise and sleep quality. Psychological resilience, also known as psychological elasticity, recovery, or adversity resistance, refers to the core psychological trait of individuals in coping with stress, setbacks, and trauma, representing an important positive psychological quality (Sisto et al., 2019). First, research indicates that physical exercise positively predicts students’ psychological resilience levels. Regular exercise and moderate-intensity activities significantly enhance psychological resilience, making them effective pathways for cultivating this trait (Qiu et al., 2025). Research emphasizes that adolescents with high exercise volumes generally exhibit higher psychological resilience. Such individuals often demonstrate greater self-control, persistence, and positive attitudes in adversity, thereby enhancing their emotional regulation and interpersonal coping abilities (McGeown et al., 2017). Physical exercise not only directly enhances adolescents’ psychological resilience but also significantly influences the development of their social–emotional competencies through partial mediating effects of this trait (Wang and Bu, 2023). This demonstrates the strong association between physical exercise and psychological resilience. Furthermore, research confirms a significant positive correlation between an individual’s psychological resilience and sleep quality (Li and Guo, 2023). From a biological perspective, psychological resilience plays a crucial role in mitigating neuroendocrine fluctuations caused by sleep deprivation, thereby promoting sleep improvement (Lo Martire et al., 2024). As a vital protective factor, psychological resilience buffers the adverse effects of negative life events, reduces depressive symptoms, and mitigates the negative impact of such events and depression on sleep quality (Wu N. et al., 2024; Wu Y. et al., 2024). Research also indicates that students with low psychological resilience in high-risk environments exhibit more pronounced emotional and behavioral issues, which often lead to the onset or exacerbation of sleep problems. Psychological resilience can also effectively mitigate the adverse effects of daily stressful life events on students’ sleep quality (Li et al., 2019). This sufficiently demonstrates that psychological resilience has been proven to be an effective predictor of sleep quality. In summary, it can be inferred that physical exercise behavior, psychological resilience, and individual sleep quality are closely interrelated. Based on this, Hypothesis 3 is proposed: Psychological resilience mediates the relationship between physical exercise and sleep quality.

### 2.4 Chain mediation effect of sense of meaning in life and psychological resilience

Existing research indicates that a sense of life meaning and psychological resilience may play significant mediating roles between physical exercise and sleep quality. As a positive psychological resource, psychological resilience shows a significant positive correlation with positive life attitudes and the pursuit of life meaning (Zheng et al., 2023). A higher sense of life meaning helps individuals develop a stable value belief system and further enhances psychological resilience through positive interpersonal connections and social support networks (Resnick, 2018). Conversely, psychological resilience improves individuals’ ability to receive and utilize social support, and reinforces the sense of life meaning through positive coping mechanisms (Afita and Nuranasmita, 2023). Thus, life meaning and psychological resilience dynamically interact during psychological adaptation. Physical exercise, as a key method for fostering positive psychological qualities, not only directly enhances life meaning but also further strengthens psychological resilience through this enhancement, thereby providing intrinsic psychological momentum for emotional regulation and stress relief. This psychological process helps explain the underlying mechanism by which physical exercise improves sleep quality.

Multiple studies provide empirical support for this causal chain. For instance, research indicates that regular physical exercise significantly enhances psychological resilience while simultaneously increasing individuals’ self-efficacy and experiences of life meaning (Jin and Dong, 2023). Scholars note that individuals with higher resilience levels demonstrate greater goal persistence and proactive coping during exercise, a process that in turn helps consolidate life meaning. Based on positive psychology theory, both life meaning and psychological resilience aid individuals in regulating emotions and alleviating psychological stress, thereby improving sleep quality (Armstrong et al., 2019; Xu et al., 2023). Longitudinal studies further indicate that enhanced life meaning significantly predicts increased psychological resilience, with this effect being more pronounced under high-stress conditions (Young et al., 2022). Conversely, individuals with higher psychological resilience exhibit better sleep quality, such as shorter sleep latency and higher sleep efficiency. This effect remains robust even after controlling for variables like age, gender, and physical health status (Cai et al., 2023). Collectively, these findings suggest that physical exercise improves sleep quality by enhancing psychological resilience through increased life meaning. Therefore, this study proposes Hypothesis 4: Life meaning and psychological resilience exert a continuous mediating role between physical exercise and sleep quality.

**Figure 1:**
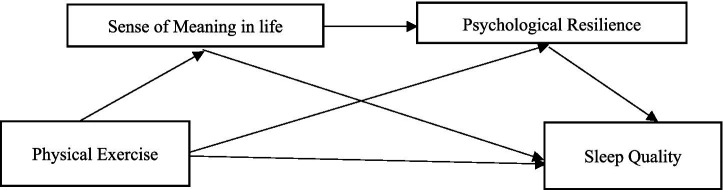
Research paradigm diagram.

In summary, scholars have identified the association between physical exercise and sleep quality among secondary school students, emphasizing the mediating and chain-mediated roles of life meaning and psychological resilience in this relationship. These findings provide theoretical justification for the hypotheses of this study. Thus, the conceptual framework of this study is established (Figure 1): (1) Examine the predictive role of physical exercise on sleep quality among secondary school students; (2) Investigate the mediating role of life meaning between physical exercise and sleep quality; (3) Investigate the mediating role of psychological resilience between physical exercise and sleep quality; (4) Test the chain mediating role of life meaning and psychological resilience in the relationship between physical exercise and sleep quality.

## 3 Research subjects and methods

### 3.1 Research subjects

This study employed stratified cluster random sampling to conduct a questionnaire survey among junior high school students in Zhaoqing City, China. First, stratification was based on school grade and class, with seventh, eighth, and ninth graders serving as separate sampling strata. Within each stratum, classes were randomly selected as survey subjects. A total of 1,628 questionnaires were distributed. After excluding invalid responses (including those with obvious patterned answers, missing key data, or incomplete information), 1,579 valid questionnaires were obtained, yielding a response rate of 96.99%. The sample age range was 12–15 years, with a mean age of 13.78 ± 0.96 years. The sample comprised 833 males and 746 females; 568 were in the first year of junior high school, 469 in the second year, and 542 in the third year. The inclusion criteria for this study’s questionnaire were: (1) Currently enrolled junior high school students aged 12–15; (2) Informed consent and voluntary participation in the study; (3) Complete questionnaire responses without obvious logical errors. Exclusion criteria were: (1) Significant data missing or key variables left blank; (2) Obvious patterned responses (e.g., selecting the same option for all questions), contradictory or unreasonable answers; (3) Failure to obtain informed consent or refusal to participate. This study was approved by the Research Ethics Committee of Zhaoqing University (No. 2025053). This study employed a cross-sectional survey formula to calculate the sample size: *n* = *Z*^2^*p(1 − *p*)/*d*^2^ (Assuming significance level *α* = 0.05, *Z* = 1.96; estimated proportion *p* = 0.5; acceptable sampling error *d* = 0.025). The calculated required sample size was 1,537.44. After adjusting for the design effect of stratified cluster sampling (DEFF = 1.03), the precise required sample size was 1,583.56. The final effective sample size was 1,579, 0.29% lower than the calculated value. This minor deviation falls within the acceptable tolerance range for sampling surveys and has a negligible impact on statistical power (primary analysis power remains >0.80). In survey methodology, minor deviations between sample size and planned size are generally considered acceptable as long as statistical power remains above the conventional threshold of 0.80 (Cohen, 2013). The literature often operationally defines “minor deviation” as a target sample size deviation of less than 5% (Groves et al., 2011). In this study, the final sample size comprised 1,579 participants, compared to the planned sample size of 1,583 participants, representing a deviation rate of Z%, which falls below the aforementioned threshold. A post-hoc power analysis based on the actual sample size revealed that the statistical power for the primary analysis remained at 0.84, indicating that the impact of the deviation is negligible (Biau et al., 2008). Therefore, the obtained sample size adequately meets the requirements of this study. Additionally, Prior to the survey, all students were informed that the questionnaire was anonymous, participation was entirely voluntary, content would be strictly confidential, and results would be used solely for scientific research. All participants received detailed explanations of the study’s purpose and procedures and signed informed consent forms. The questionnaire was administered by professionally trained undergraduate student interviewers to ensure scientific rigor and standardized data collection.

### 3.2 Measurement

#### 3.2.1 Physical exercise

The Physical Exercise Scale for Junior High School Students, revised by Wu (2016), was adopted. This scale was adapted from the Physical Exercise Commitment Intention Scale developed by Chen et al. (2006). The current scale consists of 8 items, covering two dimensions: physical exercise commitment (e.g., “It is difficult for me to withdraw from physical exercise”) and physical exercise persistence (e.g., “I maintain my physical exercise routine fairly well”). Each dimension comprises four items. A 5-point Likert scale was used, with responses ranging from “Strongly Disagree” to “Strongly Agree,” scored from 1 to 5 points, respectively. The total score indicates the participant’s level of physical exercise. Higher total scores indicate greater physical exercise levels. This scale has demonstrated high applicability among Chinese junior high school students (Jiang et al., 2018). In this study, the scale achieved an internal consistency reliability coefficient *α*of 0.94.

#### 3.2.2 Sleep quality

Sleep quality was measured using the single-item question “Over the past month, how would you rate your overall sleep quality?” from the Pittsburgh Sleep Quality Index (PSQI) developed by Buysse et al. (1989) to assess participants’ subjective sleep quality. In the original questionnaire, 1 = very good, 2 = fair, 3 = poor, 4 = very poor. For interpretability, this item was reverse-scored: 1 = very poor, 2 = poor, 3 = fair, 4 = very good, yielding a score range of 1–4. Higher scores indicate better subjective sleep quality. Previous research indicates this scale demonstrates good reliability and validity among Chinese junior high school students (Liu and Wang, 2024). In this study, the scale achieved an internal consistency reliability coefficient *α* of 0.92.

#### 3.2.3 Sense of meaning in life

Meaning in Life was assessed using the Meaning in Life Scale developed by Steger et al. (2006) and revised by Liu and Gan (2010) to measure individuals’ levels of meaning in life. The scale comprises two dimensions: Sense of Meaning in Life (e.g., “I understand the meaning of my life”) and Meaning-Seeking (e.g., “I am searching for a purpose or mission in my life”). The scale employs a 7-point Likert scale, ranging from 1 = Strongly disagree to 7 = Strongly agree. Higher scores indicate stronger perceptions or exploration of life meaning. Previous research has demonstrated the scale’s good reliability and validity among junior high school students (Lu et al., 2024). In this study, the scale’s internal consistency reliability coefficient *α* was 0.80.

#### 3.2.4 Psychological resilience

The psychological resilience subscale from the Chinese version of the Positive Adolescent Development Scale developed by Shek et al. (2007) was used. This subscale comprises three items (e.g., “When I encounter difficulties, I can find ways to solve problems”). The scale employs a 1–6 rating scale (“1” denotes “Strongly Disagree,” ‘6’ denotes “Strongly Agree”), with total scores calculated by summing items. Higher scores indicate greater psychological resilience. Previous research demonstrates this scale’s good reliability and validity among junior high school students (Wang et al., 2024). In this study, the internal consistency reliability coefficient α for this scale was 0.81.

### 3.3 Mathematical statistics methods

First, descriptive statistics were conducted on the sample data using SPSS 26.0 software to comprehensively present the basic characteristics of the research subjects. Pearson correlation analysis was employed to explore the relationships among key variables including physical exercise, sleep quality, sense of meaning in life, and psychological resilience. Second, to examine differences across gender and grade groups, independent samples t-tests and one-way analysis of variance (ANOVA) were, respectively, applied. In regression and mediation analyses, age and gender were explicitly controlled as covariates to minimize confounding effects. Third, mediation effects were examined using the PROCESS macro (Model 6) to construct a chained mediation model, analyzing the pathways through which life meaning and psychological resilience mediate the relationship between physical exercise and sleep quality. Indirect effects were estimated using 5,000 Bootstrap samples, with 95% confidence intervals reported to assess mediation significance. All statistical tests were two-tailed, with a significance level set at *p* < 0.05 to ensure statistical significance and robustness of results.

## 4 Results

### 4.1 Descriptive statistics for physical exercise, sense of meaning in life, psychological resilience, and sleep quality

Table 1 results indicate that gender differences in physical exercise, sense of life meaning, psychological resilience, and sleep quality were statistically significant (*p* < 0.001). Psychological resilience also showed statistically significant differences across grade levels (*p* < 0.001), while the other three variables did not. Sleep quality showed statistical significance in age differences (*p* = 0.003 < 0.05), while the other three variables did not. Male students scored higher than female students on average across all four variables: physical exercise, sleep quality, sense of meaning in life, and psychological resilience. Furthermore, these four variables exhibited certain patterns across different statistical measures, aiding this study in further understanding the degree of influence and interrelationships among physical exercise, sense of meaning in life, psychological resilience, and sleep quality.

**Table 1:** Descriptive statistics (*x* ± SD) for physical exercise, sense of meaning in life, psychological resilience, and sleep quality test results.

Gender	N/person	Physical exercise	Sleep quality	Sense of meaning in life	Psychological resilience
Male	833	30.61 ± 7.23	3.06 ± 0.77	47.11 ± 8.52	14.85 ± 2.79
Female	746	25.37 ± 6.39	2.91 ± 0.76	45.21 ± 8.33	13.28 ± 3.05
Overall	1,579	28.13 ± 7.33	2.99 ± 0.77	46.21 ± 8.48	14.11 ± 3.02
Gender differences (T/P)		15.184/0.000**	3.912/0.000**	4.468/0.000**	10.675/0.000**
Grade differences (F/P)		1.735/0.177	0.606/0.545	0.758/0.469	8.720/0.000**
Age differences (F/P)		0.44/0.725	4.640/0.003*	1.409/0.238	0.694/0.556

*P < 0.05, **P < 0.001 indicates significant differences in the variable across gender, age, and grade.

### 4.2 Common method bias test

This study employed Harman’s single-factor test to examine common method bias, conducting a non-rotated exploratory factor analysis on all measurement items simultaneously. Results revealed four factors with eigenvalues exceeding 1. The first factor explained 38.14% of variance, falling below the commonly accepted 40% threshold in academic research (Chen et al., 2025). This indicates that no single factor accounted for the majority of explanatory power, suggesting no substantial measurement-induced variance in the data. Thus, no severe common method bias was observed in questionnaire administration, data collection, or variable measurement in this study, ensuring high reliability of the internal validity of research conclusions.

### 4.3 Correlation analysis of physical exercise, sense of life meaning, psychological resilience, and sleep quality

Table 2 shows that physical exercise correlates significantly with sleep quality (*R* = 0.27, *p* < 0.001), sense of life meaning (*R* = 0.43, *p* < 0.001), and psychological resilience (*R* = 0.50, *p* < 0.001); Sleep quality showed significant positive correlations with sense of meaning in life (*R* = 0.27, *p* < 0.001) and psychological resilience (*R* = 0.39, *p* < 0.001);sense of meaning in life was significantly positively correlated with psychological resilience (*R* = 0.49, *p* < 0.001). These inter-variable correlations support subsequent hypothesis testing and provide a solid foundation for examining mediating effects in this study.

**Table 2:** Correlation analysis statistics for physical exercise, sense of meaning in life, psychological resilience, and sleep quality.

Variable	*M*	SD	Physical exercise	Sense of meaning in life	Psychological resilience	Sleep quality
Physical exercise	28.132	7.325	1			
Sense of meaning in life	46.211	8.481	0.428**	1		
Psychological resilience	14.110	3.020	0.504**	0.488**	1	
Sleep quality	2.989	0.770	0.268**	0.268**	0.389**	1

*N* = 1,579; **p < 0.001, same below.

### 4.4 Testing the mediating effects of physical exercise, sense of meaning in life, psychological resilience, and sleep quality

Using physical exercise as the independent variable, sense of meaning in life and psychological resilience as potential mediators, and sleep quality as the dependent variable, a mediation model was tested (PROCESS Model 6, bootstrap = 5,000). In this model, physical exercise was positively associated with sleep quality (*β* = 0.028, *p* < 0.001). Physical exercise was positively associated with sense of meaning in life (*β* = 0.517, *p* < 0.001) and with psychological resilience (*β* = 0.132, *p* < 0.001). Sense of meaning in life was positively associated with psychological resilience (*β* = 0.120, *p* < 0.001). Indirect effects via both mediators were statistically significant, with 95% bootstrap confidence intervals not containing zero. However, given the cross-sectional design, these mediation patterns should be interpreted as statistical associations rather than evidence of causal or temporal processes (Table 3).

**Table 3:** Regression analysis of the chain mediation model between physical exercise and sleep quality.

Variable	Sense of meaning in life		Psychological resilience		Sleep quality		Overall effect	
	*β*	*t*	*β*	*t*	*β*	*t*	*β*	*t*
Physical exercise	0.517	18.350**	0.132	13.434**	0.010	3.288*	0.029	10.633**
Sense of meaning in life			0.120	15.051**	0.007	2.892**		
Psychological resilience					0.079	10.793*		
R^2^	0.187		0.355		0.173		0.085	
F	90.665**		173.032**		54.962**		36.540**	

*P < 0.05, **p < 0.001.

The mediation effect analysis revealed (see Table 4) that both the chain mediation and simple mediation effects of life meaning and psychological resilience were significant. The Bootstrap 95% confidence interval for the indirect effect of physical exercise on middle school students’ sleep quality did not include zero, indicating that life meaning and psychological resilience exerted significant mediating effects between physical exercise and sleep quality (direct effect: 37.93%; indirect effect: 62.07%). This mediating effect comprised three indirect pathways: (physical exercise → sense of meaning in life → sleep quality; physical exercise → psychological resilience → sleep quality; physical exercise → sense of meaning in life → psychological resilience → sleep quality), which further validates the study’s hypotheses.

**Table 4:** Testing the chain mediating effects of sense of meaning in life and psychological resilience on physical exercise and sleep quality.

Intermediate path	Effect size	Effect weighting	95% confidence interval	
			Lower limit	Upper limit
Physical exercise → Sense of life meaning → Sleep quality	0.003	10.35%	0.009	0.06
Physical exercise → Psychological resilience → Sleep quality	0.010	34.48%	0.073	0.128
Physical exercise → Sense of life meaning → Psychological resilience → Sleep quality	0.005	17.24%	0.034	0.061
Direct effect	0.011	37.93%	0.004	0.016
Total effect	0.029		0.144	0.216

**Figure 2:**
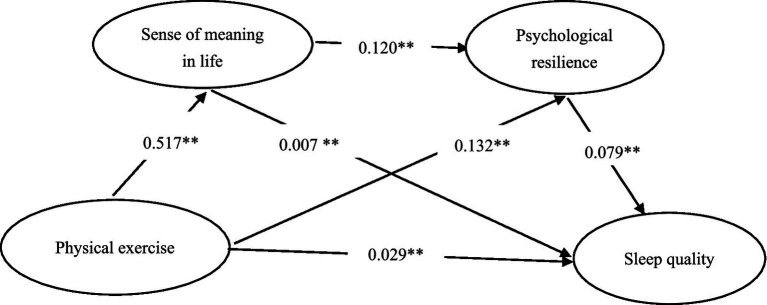
Chain mediation model of meaning in life and psychological resilience between physical exercise and sleep quality. **p < 0.001.

Given the cross-sectional nature of the data, the results cannot establish temporal sequence or causality among the variables. The mediation analyses reflect statistical associations consistent with the hypothesized pathways, but these should not be interpreted as evidence of causal mediation effects (Figure 2).

## 5 Discussion

### 5.1 Physical exercise and sleep quality

The results of this study indicate that physical exercise has a significant positive effect on sleep quality (*β* = 0.028, *p* < 0.001), consistent with existing research. Previous literature has demonstrated that daily physical exercise or physical activity is moderately to highly correlated with sleep quality, and that exercise interventions can effectively improve sleep problems (Gong et al., 2020). Therefore, Hypothesis 1 is validated. Regarding potential mechanisms, prior research suggests physical exercise improves sleep through multiple physiological pathways. For instance, physical activity elevates body temperature and facilitates efficient heat dissipation via hypothalamic regulation, thereby prolonging slow-wave sleep duration. Additionally, it may stimulate increased melatonin secretion, enhancing sleep quality (Korkutata et al., 2025; Herberger et al., 2024). Regular moderate-intensity aerobic exercise is also thought to promote neurotransmitter secretion (e.g., dopamine, endorphins, and serotonin), improve mood, alleviate stress, and thereby enhance parasympathetic activity, facilitating sleep onset and continuity (Cabral, 2025; Jerath et al., 2019). According to self-regulation theory, exercise can also reduce cortisol levels, shorten sleep onset latency, and improve sleep efficiency (Ghiasvand and Irandoust, 2024). However, it should be noted that the physiological mechanisms described above (e.g., regulation of melatonin, cortisol, and neurotransmitters) were not directly measured in this study. They represent reasonable inferences based on existing literature, and their specific pathways require further validation through physiological data collection. Future research integrating physiological monitoring techniques (e.g., hormone level testing, EEG monitoring) could empirically test these mechanistic hypotheses, thereby more precisely elucidating the causal chain linking exercise and sleep. Furthermore, from a psychological perspective, this study additionally suggests that physical exercise may indirectly enhance sleep quality by boosting psychological resilience, strengthening self-efficacy, and improving emotional regulation. Middle school students often gain a sense of accomplishment and social support through participation in physical activities, which helps alleviate psychological stress stemming from academic pressure, peer relationship tensions, or exam anxiety (Biddle and Asare, 2011; Zou et al., 2023). Positive exercise experiences can elevate self-esteem and self-identity, psychological factors significantly and positively correlated with sleep quality (Chambers, 2021). Based on cognitive-behavioral theory, physical exercise also helps students establish healthy routines and positive sleep cognitions, reducing sleep onset difficulties caused by worry and rumination (Chu et al., 2018), thereby improving sleep stability and continuity.

Notably, while the association between physical exercise and sleep quality is significant, the effect size for the variable “meaning in life” in this study is relatively weak (*β* = 0.08, *p* < 0.05). This modest effect may stem from multiple factors: First, middle school students are in a developmental stage where perceptions of life meaning remain unstable. Second, school environments or family backgrounds may weaken the direct link between life meaning and sleep. Additionally, life meaning may indirectly influence sleep through mediating factors such as emotional states or social support. Future research combining longitudinal designs and contextual analyses could further explore how developmental or environmental factors moderate the relationship between life meaning and sleep quality. In summary, consistent with prior research, these findings indicate that physical exercise promotes adolescent sleep health through multiple physiological (yet to be validated) and psychological mechanisms. However, caution is warranted when articulating physiological pathways, distinguishing existing inferences from unmeasured hypotheses. Future studies should integrate physiological monitoring and contextual analysis to yield more comprehensive and rigorous conclusions.

### 5.2 Independent mediating effect of life meaning

This study found that life meaning significantly mediates the relationship between physical exercise and sleep quality, further validating Hypothesis 2: The significant positive association between physical exercise and students’ life meaning aligns with existing research conclusions. The rationale lies in Self-Determination Theory, which posits that physical exercise satisfies three fundamental psychological needs: autonomy, competence, and relatedness. This process enhances individuals’ awareness and exploration of these core needs, thereby fostering a greater sense of life meaning (Standage and Ryan, 2020). Specifically, physical exercise not only provides students with opportunities to autonomously choose exercise forms and goals but also fosters a sense of competence through skill development and strengthens social bonds in team sports (Kao, 2019). These positive psychological experiences collectively enhance students’ awareness of life’s value and existential meaning. Research further demonstrates that adolescents engaging in moderate-to-vigorous physical activity score significantly higher on life meaning than their sedentary peers. This occurs because exercise-induced endorphin release elevates positive emotions, which form a crucial cognitive foundation for meaning perception (Chen and Nakagawa, 2023; Barca, 2025). Additionally, scholars note a correlation between life meaning and sleep quality. Individuals with higher life meaning tend to exhibit more stable emotional states and lower psychological stress levels in daily life, thereby reducing the likelihood of insomnia (Ge, 2025). From a psychological mechanism perspective, life meaning enhances resilience in coping with life challenges, enabling better emotional regulation when facing stress and anxiety, which contributes to maintaining high sleep efficiency (Zhu et al., 2025). Students with greater life meaning typically maintain healthier lifestyle habits and schedules. This self-management capacity supports stable sleep rhythms, further improving sleep quality (Berger, 2024). Finally, in summary, physical exercise improves sleep not only through direct physiological mechanisms (such as increased endorphin secretion and reduced cortisol levels) but may also indirectly influence sleep quality by enhancing life meaning. Research demonstrates that the positive emotions and sense of accomplishment promoted by exercise strengthen individuals’ life meaning, which in turn improves sleep quality by reducing negative thoughts before bedtime and increasing relaxation levels (Ye et al., 2022; Armstrong et al., 2019). Thus, this study supports the view that life meaning plays an independent and significant mediating role between physical exercise and sleep quality, providing a viable theoretical basis for mental health interventions and sleep improvement programs among secondary school students.

### 5.3 Independent mediating effect of psychological resilience

This study found that psychological resilience significantly mediates the relationship between physical exercise and sleep quality, further validating Hypothesis 3: Psychological resilience exhibits a significant independent mediating effect between physical exercise and sleep quality, explicitly supporting the transmission pathway “Physical Exercise → Psychological Resilience → Sleep Quality.” This aligns with prior research indicating that regular physical exercise significantly enhances students’ psychological resilience. The underlying mechanisms are twofold: First, according to the stress buffering hypothesis, exercise reduces physiological and psychological stress responses, thereby providing psychological resources for coping with stress and challenges (Klaperski and Fuchs, 2021). Physiologically, moderate-to-vigorous physical activity regulates cortisol secretion rhythms, promotes brain-derived neurotrophic factor (BDNF) release, and enhances prefrontal cortex function. These changes bolster emotional regulation and cognitive flexibility, thereby strengthening psychological resilience (Rawliuk, 2024). Psychologically, the sense of accomplishment derived from achieving goals and improving skills during exercise enhances self-efficacy, which is closely associated with greater psychological resilience (Kim et al., 2023). Secondly, scholars also note a significant positive correlation between psychological resilience and sleep quality. Individuals with high psychological resilience tend to adopt proactive coping strategies—such as problem-solving and emotional regulation—when facing life stressors, rather than passive avoidance or rumination. This approach helps reduce pre-sleep anxiety and cognitive hyperarousal (Wu N. et al., 2024; Wu Y. et al., 2024). One study demonstrated that adolescents with higher psychological resilience reported shorter sleep latency, fewer nighttime awakenings, and longer total sleep duration, suggesting psychological resilience may directly improve sleep efficiency by reducing negative emotional interference (Wang et al., 2020). Furthermore, psychological resilience is closely linked to mental health levels. Enhancing resilience can lower depression, anxiety, and stress levels—psychological states that are significant risk factors for sleep disorders (Song et al., 2021). Therefore, improving psychological resilience is believed to enhance students’ sleep quality and stability. Finally, in summary, physical exercise not only directly improves sleep through physiological and psychological mechanisms but also indirectly influences sleep quality by enhancing psychological resilience, forming a mediating pathway: “physical exercise → psychological resilience → sleep quality.” This aligns with resource conservation theory, which posits that physical exercise, as a resource acquisition behavior, not only directly creates positive physiological and psychological states but also accumulates psychological resilience—a core psychological resource—thereby reducing the disruptive impact of stress on sleep (Guo, 2025). Research also indicates that adolescents who regularly engage in physical exercise exhibit significantly higher psychological resilience levels than low-activity groups, and high resilience levels effectively predict better sleep quality in subsequent measurements (Meng et al., 2025). Thus, psychological resilience exerts a significant and independent mediating effect between physical exercise and sleep quality. This finding holds important practical implications for adolescent mental health promotion and the development of sleep intervention strategies.

### 5.4 Chain mediation effect of life meaning and psychological resilience

This study delves into the chain-mediated pathway of “physical exercise → sense of meaning in life → psychological resilience →sleep quality,” revealing the crucial role of the synergistic interaction between sense of meaning in life and psychological resilience in improving sleep quality through physical exercise (further validating Hypothesis 4). This aligns with prior research indicating a significant positive correlation between sense of meaning in life and psychological resilience. The underlying mechanism can be explained by the Resource Gain Spiral Theory, which posits that acquired primary psychological resources facilitate the accumulation of secondary psychological resources, creating sustained positive feedback (Jiang et al., 2023). In the context of physical exercise, life meaning—as a distal cognitive resource—provides individuals with clear life goals and value guidance. This equips them with stronger motivation and direction when confronting stressful situations (Hooker et al., 2018). This goal orientation not only enhances confidence and persistence in adversity but also further fosters psychological resilience by amplifying positive emotional experiences and self-efficacy. Additional research indicates that individuals with high life meaning are more likely to employ adaptive coping strategies such as problem-solving and positive reappraisal when facing stressful events (Bondarchuk et al., 2024), which constitute the core components of psychological resilience. Thus, a sense of life meaning not only provides the cognitive and emotional foundation for enhancing psychological resilience but may also foster more stable emotional regulation and stress resistance through the continuous accumulation of psychological resources. Second, in this study, physical exercise as an external behavioral intervention not only directly improves physical health but also indirectly enhances sleep quality by stimulating a sense of life meaning and thereby strengthening psychological resilience. According to self-determination theory, physical exercise satisfies three fundamental psychological needs—autonomy, competence, and relatedness—thereby elevating students’ sense of life meaning. This leads them to perceive exercise as a pathway to self-actualization and social connection (Zhang et al., 2022). Enhanced life meaning further strengthens psychological resilience in adversity, reducing cognitive hyperarousal and pre-sleep anxiety during stressful events, thereby optimizing sleep architecture (Chen, 2023). The social support networks provided by team or social sports lower physiological stress levels (e.g., by reducing cortisol secretion) and stabilize emotional states (McKimmie et al., 2020; Martín-Rodríguez et al., 2024). Collectively, the chain reaction of positive psychological resource transmission—“physical exercise → sense of meaning in life → psychological resilience → sleep quality”—not only reveals the relay mechanism between hierarchical psychological resources but also provides a more systematic theoretical framework and practical pathway for adolescent sleep intervention than single psychological variables alone.

## 6 Practical implications

This study not only expands the theoretical understanding of the mechanisms underlying adolescent sleep quality but also provides scientific evidence for school health education and psychological interventions in practice. By validating the mediating pathway “physical exercise → sense of meaning in life → psychological resilience → sleep quality,” it reveals the hierarchical beneficial effects of physical exercise on adolescents’ psychological resources, enriching cross-system integration models in sleep research. At the application level, this study organically integrates self-determination theory with resource conservation theory, proposing that physical exercise not only satisfies adolescents’ needs for autonomy and competence but also buffers stress by enhancing psychological resilience. This offers a new theoretical perspective for understanding the multistage, progressive influence mechanism between exercise and sleep.

Addressing the profound physical and psychological transformations during adolescence, this study recommends schools implement structured, sustainable exercise interventions in physical education and health curricula: (1) Activity Types: Prioritize aerobic exercises (e.g., middle/long-distance running, jump rope, aerobics), ball sports (e.g., basketball, volleyball), and mind–body activities (e.g., yoga, tai chi), balancing enjoyment and accessibility; (2) Exercise Intensity: Maintain moderate intensity (heart rate at 60–75% of maximum during activity) to boost physical vitality while avoiding excessive fatigue that impairs learning; (3) Frequency and Duration: Engage in exercise at least 3–5 times weekly for 30–45 min per session. Incorporate activities into physical education classes, recess exercises, and campus morning runs, supplemented by after-school interest-based sports clubs; (4) Family involvement: Parents may organize weekend activities like outdoor cycling, family walks, or home fitness challenges. Incorporate healthy habits into household routines, such as limiting screen time 1 h before bed and practicing relaxation stretches.

Additionally, schools can integrate “sense of life meaning” and “psychological resilience” cultivation into physical education through mental health courses and class meetings. By incorporating goal-setting, teamwork, and achievement feedback, schools can enhance the emotional support and sense of accomplishment students gain from physical activities. Coordinated family-school interventions will help adolescents effectively manage academic stress and emotional distress, thereby optimizing sleep quality and overall mental health. In summary, these findings not only provide crucial evidence for adolescent health behavior theory but also offer a robust theoretical foundation and practical guidance for developing actionable, scalable, and sustainable strategies to promote physical and mental well-being.

## 7 Limitations and future directions

This study has made some progress in revealing the mediating and path mechanisms among physical exercise, sense of meaning in life, psychological resilience, and sleep quality. However, several limitations remain that require improvement and expansion in future research.

### 7.1 Limitations of measurement methods

This study employed self-reported questionnaires to measure sleep quality, which is susceptible to social desirability bias and limitations of self-perception. This may compromise the objectivity and validity of the measurements. To enhance measurement rigor, future studies may incorporate multimodal objective indicators such as: – Wearable devices monitoring deep sleep duration – Polysomnography analyzing sleep stages and EEG characteristics – Salivary/serum melatonin level detection Integrating subjective and objective data would strengthen the reliability and validity of findings.

### 7.2 Limitations in study design and causal inference

This cross-sectional study design can only reveal correlations between variables and cannot directly infer causal directionality. For example, individuals with high psychological resilience may be more inclined to maintain exercise habits, or their resilience may have been enhanced by long-term exercise—the temporal sequence between these factors requires further clarification. Future studies may employ longitudinal tracking to investigate the long-term effects of exercise on sleep quality across different stages, or conduct randomized controlled trials (RCTs) to validate causal relationships. Concurrently, dynamic monitoring of changes in distinct sleep stages before and after exercise could help determine the causal impact of specific exercise patterns.

### 7.3 Inadequate control for confounding factors

This study did not sufficiently control for potential confounding variables such as academic stress, family economic status, and social support, which may simultaneously influence both psychological resilience and sleep quality. Future research should incorporate statistical methods like stratified regression analysis, propensity score matching, or multilevel linear models (HLM) to more effectively control for multilevel confounding factors during modeling, thereby enhancing model interpretability and external validity.

### 7.4 Model robustness and hypothesis testing

Methodologically, further reporting and examination of model robustness should be conducted, including: whether model fit indices (such as CFI, TLI, RMSEA, etc.) meet standards; whether assumptions (such as normality, homoscedasticity, linear relationships, etc.) are satisfied; and testing model stability across different samples or subgroups (multigroup analysis). This will enhance the credibility and replicability of the results.

### 7.5 Sample and generalizability

The current study sample is limited to junior high school students with a narrow age range. Caution is warranted when generalizing findings to other age groups (e.g., elementary school students, high school students, college students). Future research should expand to adolescent populations across different ages, genders, regions, and cultural backgrounds to further compare differences in physical exercise, psychological resilience, and sleep mechanisms across samples.

### 7.6 Future research directions and practical applications: future studies may explore the following areas

First, differentiate exercise types and dose effects by comparing aerobic, resistance training, and mind–body exercises (e.g., yoga, tai chi) in terms of intensity, frequency, and duration to identify the “optimal exercise prescription” for sleep improvement. Second, investigate the influence of circadian rhythms and exercise timing, examining differential effects of morning versus evening workouts and sleep improvement among different chronotypes (day/night). Third, analyze physiological mechanisms through polysomnography and EEG analysis, exploring physiological pathways such as extended slow-wave sleep and elevated melatonin secretion. Fourth, examine the mediating and moderating roles of psychosocial variables. Incorporate factors such as perceived stress, emotional regulation, academic burden, and social support to explore their role in the relationship between physical exercise and sleep improvement. Fifth, translate findings into policy and intervention practices. Apply research outcomes to optimize school physical education curricula, develop adolescent sleep health education programs, and design exercise intervention protocols. Establish a scalable, exercise-based model for promoting school sleep health, providing evidence-based support for the Healthy China strategy.

## 8 Conclusion

This study examined the mechanism through which physical exercise influences sleep quality in junior high school students. Results indicate that physical exercise not only directly improves adolescents’ sleep quality but also exerts indirect effects by enhancing life meaning and psychological resilience. Life meaning and psychological resilience demonstrated significant mediating effects in this relationship, revealing the central role of psychological resources in the association between exercise and sleep. This study is the first in adolescent mental health research to integrate meaning in life and psychological resilience into a unified model of the relationship between physical exercise and sleep quality. It constructs a novel cross-system theoretical framework, offering new perspectives and theoretical foundations for explaining the exercise-sleep relationship. These findings not only enrich the theoretical system of adolescent sleep research but also provide empirical support and practical guidance for school physical education curriculum design, mental health education, and sleep quality interventions.

However, the cross-sectional data-based empirical analysis in this study has certain limitations, making it difficult to fully reveal the dynamic evolution and causal direction between variables. Future research should further validate the causal relationships among physical exercise, psychological resources, and sleep quality through longitudinal tracking or experimental designs, and explore the stability of these mechanisms across different age groups, genders, and sociocultural contexts.

## Data Availability

The original contributions presented in the study are included in the article/supplementary material, further inquiries can be directed to the corresponding author.
